# *In vitro* Mixed Biofilm of *Streptococcus suis* and *Actinobacillus pleuropneumoniae* Impacts Antibiotic Susceptibility and Modulates Virulence Factor Gene Expression

**DOI:** 10.3389/fmicb.2020.00507

**Published:** 2020-04-07

**Authors:** Yang Wang, Shenglong Gong, Xiao Dong, Jinpeng Li, Daniel Grenier, Li Yi

**Affiliations:** ^1^College of Animal Science and Technology, Henan University of Science and Technology, Luoyang, China; ^2^Key Laboratory of Molecular Pathogen and Immunology of Animal of Luoyang, Luoyang, China; ^3^Groupe de Recherche en Écologie Buccale, Faculté de Médecine Dentaire, Université Laval, Quebec, QC, Canada; ^4^College of Life Science, Luoyang Normal University, Luoyang, China

**Keywords:** *Streptococcus suis*, *Actinobacillus pleuropneumoniae*, mixed biofilm, antibiotic susceptibility, swine

## Abstract

*Streptococcus suis* (*S. suis*) and *Actinobacillus pleuropneumoniae* (*A. pleuropneumoniae*) are primary swine pathogens that have been frequently co-isolated from pigs suffering from severe respiratory disease. The purpose of this study was to investigate the biological impacts of the interactions between *S. suis* and *A. pleuropneumoniae*. A single- and dual-species culture model was established *in vitro* via *S. suis* HA9801 (serotype 2) and *A. pleuropneumoniae* CVCC265 (serotype 1). The single or mixed biofilms were imaged by confocal laser scanning microscopy. The biomass and viable cells in biofilms were quantified by crystal violet staining and determination of colony-forming units. The antibiotic susceptibility was determined by a microdilution broth method. The differences in gene transcription in pure- or mixed-species biofilms of *S. suis* and *A. pleuropneumoniae* was evaluated by quantitative PCR. *S. suis* and *A. pleuropneumoniae* formed two-species biofilms when co-cultured *in vitro*. When co-cultured with *S. suis*, biofilm formation by *A. pleuropneumoniae* was significantly increased with the absence of NAD that is necessary for the growth of *A. pleuropneumoniae*. Moreover, compared with monocultures, the antibiotic resistance of *S. suis* and *A. pleuropneumoniae* was both enhanced in the co-culture model. When grown in dual-species biofilms, for *A. pleuropneumoniae*, genes associated with virulence factors, including exotoxins and adhesins, were significantly upregulated. For *S. suis*, virulence factor-related genes *cps2*, *gdh*, *mrp*, and *sly* were highly induced. These results suggest that the interspecies interactions between *S. suis* and *A. pleuropneumoniae* may be cooperative under specific conditions and may play an important role in the disease progression and persistent infection.

## Introduction

Respiratory diseases constitute the most important health issues affecting the swine industry worldwide and are often referred to as the porcine respiratory disease complex (PRDC) (Qiao et al., 2011). In general, PRDC causes lung lesions, which, in turn, results in substantial economic losses and impaired animal welfare (Fablet et al., 2012). PRDC is multifactorial in nature and is triggered by mixed infections involving primary and secondary pathogens (Cheong et al., 2017). Clinically, *Streptococcus suis* (*S. suis*) and *Actinobacillus pleuropneumoniae* (*A. pleuropneumoniae*) are two important respiratory pathogens frequently detected in the PRDC (Opriessnig et al., 2011). In recent years, there have been many cases of mixed infections associated with the presence of *S. suis* and *A. pleuropneumoniae* in China. It is more than likely that the different bacteria or viruses coexisting in PRDC interact and exacerbate the pathogenesis of the disease. However, few research groups investigated the interactions between *S. suis* and *A. pleuropneumoniae*, except for a study reporting that these two bacterial species form polymicrobial biofilms *in vitro* (Ramirez-Castillo et al., 2018).

The formation of biofilm is considered as a survival strategy for bacterial pathogens. Bacteria in biofilms are embedded in an extracellular matrix, which reduces their susceptibility to antibiotic and host immune system (Vuotto and Donelli, 2019). This is a major reason why biofilm-related infections are difficult to eradicate. During a chronic infection, bacteria are able to switch between planktonic and biofilm lifestyle (Kathju et al., 2009; Lister and Horswill, 2014). The key role of biofilms have been confirmed in many diseases such as endocarditis, periodontitis, and otitis media (Kobayashi et al., 2005). Multi-species biofilms may be the most significant lifestyle of microbes *in vivo* and bacteria in such polymicrobial biofilms are more difficult to eradicate (Wang et al., 2018). In multi-species biofilms, microorganisms will enhance and ensure their survival and reproduction through communication, competition, or cooperation (Yang et al., 2011). In the present study, we hypothesized that there are synergistic interactions between *S. suis* and *A. pleuropneumoniae* in dual-species biofilms, resulting in enhanced biofilm formation ability, enhanced antibiotic resistance, and upregulated virulence factor gene expression.

## Materials and Methods

### Bacterial Strains and Growth Conditions

*S. suis* HA9801 (SS) was isolated from a diseased pig in Jiangsu Province, China, and identified to be *S. suis* serotype 2. All experiments in this study were approved by the Experimental Animal Monitoring Committee of Henan University of Science and Technology and carried out accordingly. *A. pleuropneumoniae* CVCC 265 (APP) was purchased from China Veterinary Culture Collection Center (CVCC). *S. suis* was grown in tryptic soy broth (TSB) or plated on TSA. *A. pleuropneumoniae* was cultivated in the same media supplemented with 0.1 μg/mL of β-nicotinamide-adenine-dinucleotide (NAD). Bacterial cultures were incubated at 37°C. All experiments described below were replicated in biological triplicate.

### Planktonic Growth Assays of Mono- and co-Cultures

Bacterial colonies of *S. suis* and *A. pleuropneumoniae* were inoculated separately in solid media and grown overnight to mid-exponential phase in TSB + 0.1% NAD medium. The bacterial cultures were diluted in fresh medium to obtain an optical density at 660 (OD_660_) of 0.03. Mono-culture of *S. suis* or *A. pleuropneumoniae* and co-culture of mixture (1:1 ratio) were inoculated in TSB + 0.1% NAD medium for 24 h. Samples were taken at 0, 2, 4, 6, 8, 10, 12, and 24 h, serially diluted in sterile PBS, and then plated on TSA or TSA + 0.1% NAD medium to discriminate *S. suis* or *A. pleuropneumoniae*, respectively, and the viable cell counts were enumerated.

For the mixed culture, the Competitive Index (CI) was calculated according to the formula: (*A. pleuropneumoniae*/*S. suis*)_output_/(*A. pleuropneumoniae/S. suis*)_input_. The output and input samples were assessed by plating onto selective medium at different time points, respectively. The Relative Increase Ratio (RIR) was similar to CI, and was calculated from the corresponding growth results obtained from single cultures of each strain (Macho et al., 2007). A positive CI value suggests a competitive advantage for *A. pleuropneumoniae* and vice versa. Only CIs that are statistically different from the RIRs at the same growth stages can be recognized as the result of prominent competition between species (Macho et al., 2007).

### Biofilm Formation by Single and Mixed Cultures

Biofilm production was quantified by crystal violet staining as described previously (Bragonzi et al., 2012). Briefly, overnight cultures of *S. suis* and *A. pleuropneumoniae* were diluted 1/100 in fresh TSB broth supplemented with NAD, and inoculated individually or at several different ratios in 96-well plates. After 24 h incubation, the medium and planktonic bacteria were removed, and each well was gently washed twice with sterile PBS. Methanol was used to fix the attached bacteria for 15 min. The plates were then air-dried and the biofilms were stained with crystal violet (0.1%). After 20 min, the excess dye was discarded and plates were washed twice with sterile PBS prior to adding 200 μl of 95% ethanol to the wells to dissolve the biofilms. The optical density at 620 nm (OD_620_) was measured using a microplate reader.

Biofilms were prepared in the 96-well plates as described above. Biofilms were washed twice with sterile PBS, and then bacteria were detached and homogenized in 100 μl of sterile PBS by weak sonication for 4 min. TSA or TSA + 0.1% NAD medium was used to discriminate *S. suis* or *A. pleuropneumoniae*, respectively, and then viable cells were enumerated (Chan et al., 2017).

### Confocal Laser Scanning Microscopy

Chamber slides were inoculated with bacterial suspension (*S. suis* alone, *A. pleuropneumoniae* alone and combination in 1:1 ratio) for 24 h at 37°C. The slides were washed twice with PBS to remove the medium and unattached bacteria. Samples was stained with SYTO 9 solution following the manufacturer’s instructions from LIVE/DEAD^TM^ BacLight^TM^ Bacterial Viability Kit (Thermo Fischer Scientific, Inc., Waltham, MA, United States), and then washed with PBS (Tawakoli et al., 2013). Biofilms were observed using a Zeiss LSM800 CLSM (Carl Zeiss, Jena, Germany).

### Antibiotic Susceptibility Testing

The *in vitro* antibiotic susceptibility of *S. suis* and *A. pleuropneumoniae* was determined by a twofold dilution method in microplates following the guidelines of Clinical and Laboratory Standards Institute. Antibiotic serial dilutions were made up in culture medium, and 100 μl was transferred to the wells. Overnight cultures of *S. suis* and *A. pleuropneumoniae* were diluted at 1:100 with TSB contain in 0.1% NAD, and 100 μl of *S. suis*, *A. pleuropneumoniae*, or combination in 1:1 were added to 96-well plates and incubated at 37°C for 24 h. The minimum inhibitory concentration (MIC) values were determined by reading the optical density and visual observation of the turbidity.

The minimum biofilm eradication concentration (MBEC) values were also determined. Biofilms were prepared in the 96-well plates as described above. Wells were washed twice with PBS and antibiotic serial dilutions made up in TSB containing 0.1% NAD were transferred (200 μl) to the wells. The plates were incubated for another 24 h. The MBEC values were determined by plating samples on culture medium plates.

### Virulence Factor Gene Expression in Mixed Biofilm

Real-time PCR was used to assess the relative expression of virulence factor genes in *A. pleuropneumoniae* or *S. suis* when grown alone or in mixed biofilms. Biofilms were prepared in 24-well plates as described above, washed twice with PBS, detached by sonicating, and then collected. RNA was extracted by the Trizol method (Pompilio et al., 2015). gDNA removal and cDNA synthesis were performed using PrimeScript^TM^ RT reagent Kit with gDNA Eraser (TaKaRa) according to the manufacturer’s protocol. Real-time PCR assay was performed with TB Green^®^ Premix Ex Taq^TM^ (TaKaRa) following the manufacturer’s instructions. The primers used are listed in Table 1. The 16S rRNA was used as the house-keeping gene for normalization. Relative expression levels were determined by the (ΔΔCt) method.

**TABLE 1:** The primers used in this study for qRT-PCR.

**Primer**	**Forward primer**	**Reverse primer**
**APP**		
*16S rRNA*	GGAGCTTGCTTTCTTTGCCGACG	TAACCTTGCGGCCGTACTCCC
*Apx-I*	TTGAAGCGGAGAAACAGCTT	TGACCGACCTCGATAAAACC
*Apx-II*	GGTCAAGGAAAT GGAGTTCAAGAT	GCTAGTTTTT GCAATGTCCAA
*afuB*	TGGTTTTTAACGAACTGCCTTT	CTTTAATGATGCGCCAATGT
*hgbA*	CGGATCCGTTTAGCTTCTTG	TAATGCGGCTTCTTTCGTCT
*Pga*	GATAAAGCAAGCCAG TTCTTAGGT	GCTGTTTGATGAG AAATACCGA
*Apa-I*	TTGCAGCAGGTGACGTGAA	TCGCTGACCGCGTATAATT
**SS**		
*16S rRNA*	GTTGCGAACGGGTGAGTAA	TCTCAGGTCGGCTATGTATCG
*cps2*	ATTGGTAGGCACTGTCGTTGGTC	AGAACTTAGCATTGTTGCGGTGG
*fbps*	AACCATCTTGCCAGGCTCCAC	CAGTTCAGAAGCCGTATCCCGAC
*gdh*	CACCTTTACCACCGCCGATTG	GGAAATGTTCAAGTCAACCGTGG
*mrp*	CAGGTAACATCAGAATCACCA CTTTT	AAGTTTTGTTTGAGCATCCT CTATAGC
*sly*	TCATTCAGGTGCTTATGTTGCG	GAAGA TTGCG AGCAT TTCCT GG

### Statistical Analysis

GraphPad Prism version 7.0 (GraphPad Software, San Diego, CA, United States) was used for data analysis. Results were obtained from three independent experiments and all values were expressed as means ± standard deviation. Differences between mean values were evaluated by Student’s *t*-test. *P*-value of 0.05 or less was considered statistically significant.

## Results

### Competition Between *S. suis* and *A. pleuropneumoniae* in Planktonic co-Cultures

**FIGURE 1:**
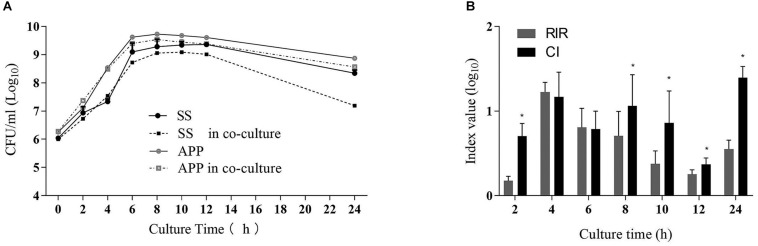
Single- and dual-species planktonic growth kinetics and competition index values. *S. suis* and *A. pleuropneumoniae* were inoculated in TSB + 0.1% NAD medium for 24 h in mono and mixed cultures. Growth curves were monitored by colony-forming unit counts (CFU) after plating on TSA or TSA + 0.1% NAD medium to discriminate *S. suis* or *A. pleuropneumoniae* respectively. **(A)** Growth curves of *S. suis* in single culture (SS) and in co-culture with *A. pleuropneumoniae* (SS in co-culture). Growth curves of *A. pleuropneumoniae* in pure culture (APP) and in co-culture with *S. suis* (APP in co-culture). **(B)** Competitive index (CI) and Relative Increase Ratio (RIR) of *S. suis* and *A. pleuropneumoniae* obtained from single culture and co-culture were calculated as described in section Materials and Methods. Error bars represent standard deviation of three separate assays. * *p* < 0.05.

To study the interactions between *S. suis* and *A. pleuropneumoniae* in planktonic co-cultures, the growth curves of single and mixed cultures were compared, and the results are shown in Figure 1A. The kinetics analysis showed that the growth of *S. suis* in mixed culture was negatively affected from 8 to 24 h, while *A. pleuropneumoniae* growth was not clearly affected when co-cultured with *S. suis*. To further estimate the differences in growth kinetics between *S. suis* and *A. pleuropneumoniae* in single or mixed cultures, CI and RIR indexes were calculated. As shown in Figure 1B, a positive CI index of *A. pleuropneumoniae* versus *S. suis* was always observed, indicating a competitive advantage for *A. pleuropneumoniae* over *S. suis* in co-cultures. The CI was significantly higher than the RIR between 8 and 24 h (*p* < 0.05), suggesting a noticeable negative effect of *A. pleuropneumoniae* on *S. suis* growth.

### Formation of Mixed Biofilm of *S. suis* and *A. pleuropneumoniae*

**FIGURE 2:**
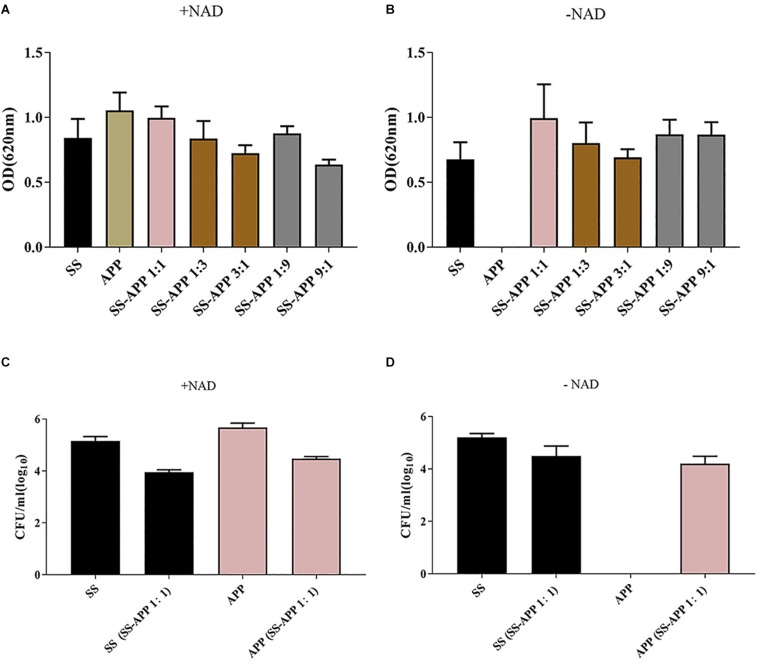
Biofilm formation by *S. suis* and *A. pleuropneumoniae* in mono and mixed cultures. Single or mixed biofilms formed by *S. suis* and *A. pleuropneumoniae* in TSB media **(A)** or in TSB + 0.1% NAD medium **(B)** were quantified by crystal violet staining. The colony-forming units (CFU) for *S. suis* and *A. pleuropneumoniae* in single or mixed biofilms were determined in TSB media **(C)** or in TSB + 0.1% NAD medium **(D)**.

Biofilm formation by *S. suis* and *A. pleuropneumoniae* in single and dual culture in 96-well plate was assessed by crystal violet staining and viable count, and the results are shown in Figure 2. Both *S. suis* and *A. pleuropneumoniae* formed important biofilms when grown in TSB supplemented with NAD (Figure 2A). However, in the absence of NAD, *A. pleuropneumoniae* couldn’t form biofilms (Figure 2B). Under favorable growth conditions(supplement with NAD)for *A. pleuropneumoniae*, mixed biofilms with *S. suis* were formed (Figure 2C). Moreover, based on results of crystal violet staining and determination of CFU, *A. pleuropneumoniae* was able to grow and form a dual-species biofilm without the addition of NAD when grown in the presence of *S. suis* (Figure 2D).

**FIGURE 3:**
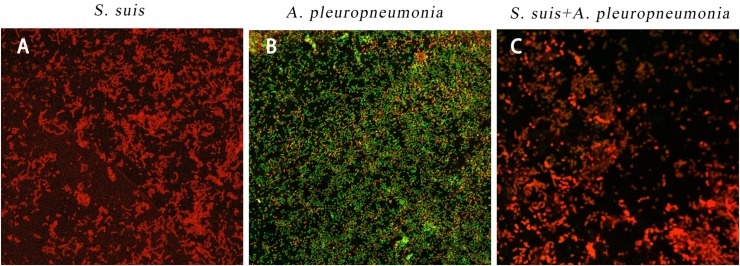
Biofilm formation of single and mixed species imaged by confocal laser scanning microscopy at × 63 magnification after 24 h inoculation. *S. suis* alone **(A)**, *A. pleuropneumoniae* alone **(B)**, or *S. suis* and *A. pleuropneumoniae* together **(C)**.

By using confocal laser scanning microscopy (Figure 3), it could be confirmed that both *S. suis* and *A. pleuropneumoniae* were able to form robust single- and dual-species biofilms *in vitro*.

### Antibiotic Susceptibility

The *in vitro* antibiotic susceptibility of *S. suis* and *A. pleuropneumoniae*, individually and in combination to several antimicrobial drugs, was determined (Table 2). For tylosin tartrate, when in co-culture with the other, the MBEC of *S. suis* increased from 1.25 to 80 μg/ml, and the MBEC of *A. pleuropneumoniae* increased from 40 to 80 μg/ml. For tilmicosin, when in co-culture, the MIC and MBEC of *S. suis* or *A. pleuropneumoniae* were twice as high as that in pure culture. In general, *S. suis* and *A. pleuropneumoniae* in mixed biofilms showed increased resistance to tylosin tartrate and tilmicosin with higher values of MBEC in comparison to single-species biofilms.

**TABLE 2:** *In vitro* susceptibility of pure culture and co-culture of *S. suis* and *A. pleuropneumoniae* in planktonic growth and biofilm to seven clinically relevant antibiotics.

**Antibiotic**	***Streptococcus suis***	***Actinobacillus pleuropeumoniae***	***S. suis* + *A. pleuro- peumoniae***
**Tylosin tartrate**			
MIC	<0.3125	20	20
MBEC	1.25	40	80
**Gentamicin**			
MIC	10	10	10
MBEC	10	10	10
**Amoxicillin**			
MIC	<0.3125	160	>160
MBEC	0.625	160	>160
**Apramycim**			
MIC	40	80	80
MBEC	40	80	>160
**Tilmicosin**			
MIC	5	5	10
MBEC	80	80	160
**Spectinomycin**			
MIC	40	80	80
MBEC	80	80	160
**Fosfomycin**			
MIC	80	20	20
MBEC	>160	40	>160

### Differential Gene Expression in Mono- and Dual-Species Biofilms

**FIGURE 4:**
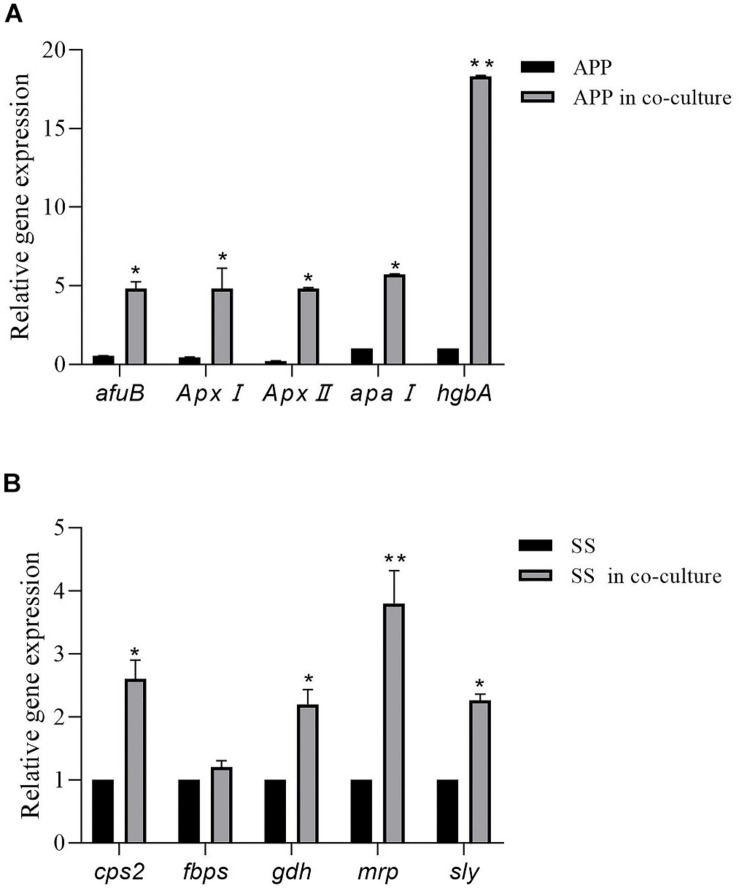
Relative expression levels of *A. pleuropneumoniae* virulence factor genes (*ApxI*, *ApxII*, *afuB*, *apaI*, and *hgbA*) in mixed biofilm **(A)**. Relative expression levels of *S. suis* virulence factor genes (*cps2*, *fbps*, *gdh*, *mrp*, and *sly*) in mixed biofilm **(B)**. Error bars represent standard deviation of three separate assays. * *p* < 0.05; ** *p* < 0.01.

As shown in Figure 4, in comparison with the single species biofilms, the virulence factor genes in the two-species biofilms were overall upregulated. For *A. pleuropneumoniae*, compared with mono-species biofilms, *ApxI* and *ApxII*, codifying for exotoxin, were upregulated by 10.65- and 22.58-fold, respectively, *afu*B involved in biofilm formation was upregulated by 8.69-fold, *apaI* associated with adhesin was upregulated by 5.73-fold, and *hgb*A involved in iron uptake was upregulated by 18.3-fold (Figure 4A). For *S. suis*, virulence factor related genes *cps2*, *gdh*, *mrp*, and *sly* were significantly upregulated by 2. 61-, 2. 23-, 3. 81-, and 2.26-fold, respectively, with no statistical differences of *fbps* gene (Figure 4B). The results may indicate that co-culture may modulate the bacterial virulence in mixed biofilm.

## Discussion

Polymicrobial respiratory diseases remain a major threat in the swine industry worldwide. Pathogens associated with PRDC include swine influenza virus (SIV), porcine circovirus type 2 (PCV2), porcine reproductive and respiratory syndrome virus (PRRSV), *S. suis*, *A. pleuropneumoniae*, *Mycoplasma hyopneumoniae*, and *Haemophilus parasuis*. Bacterial–viral co-infections were reported to exacerbate the pathogenicity (Opriessnig et al., 2011). For instance, co-infections of *M. hyopneumoniae* and SIV lead to the exacerbation of the clinical signs (Thacker et al., 1999), and during the co-infection of PRRSV and *S. suis*, the virulence of PRRSV was enhanced by *S. suis* and PRRSV increased the susceptibility of pigs to *S. suis* infection (Thanawongnuwech et al., 2000). *S. suis* and *A. pleuropneumoniae* are two important pathogens associated with PRDC, and their co-occurrence in the same site of infection has been frequently reported (Opriessnig et al., 2011), although the interactions between them and the host during coinfection have not been previously investigated. The present study examined the interactions between *S. suis* and *A. pleuropneumoniae* in planktonic and biofilm cultures, and specifically explored whether these interactions provide greater fitness than single cultures, which is helpful to better understand the possible role of coinfection in the pathogenesis.

Studies on microbial interactions under planktonic growth conditions have shown that species can coexist with other microbes competing in the same repository through multiple mechanisms (Hibbing et al., 2010). It was reported that the consumption of limited nutriments may impact the interactions process between species in co-culture models (Sibley et al., 2008). We demonstrated that *S. suis* has no effect on the growth of *A. pleuropneumoniae* in planktonic culture, while a significant negative impact of *A. pleuropneumoniae* on *S. suis* growth was found during the stationary phase of bacterial growth.

When grown in mixed cultures, the exoproducts of *A. pleuropneumoniae* may be the disadvantageous cause of *S. suis* in planktonic bacteria. The CI and RIR indicated that *A. pleuropneumoniae* surpasses *S. suis* in exponential and stationary phases of bacterial growth. The competition for limited nutrients and space may lead to the antagonistic effects among microorganisms within a community (Harrison, 2007).

Crystal violet staining and colony-forming unit results showed that both *S. suis* and *A. pleuropneumoniae* formed strong biofilms when grown in single or mixed culture, and the CSLM images provided additional evidence. Interestingly, *A. pleuropneumoniae* was able to grow in the absence of NAD when co-cultured with *S. suis.* Our results confirm that the presence of *S. suis* promoted *A. pleuropneumoniae* biofilm growth under a hostile condition for *A. pleuropneumoniae* (without NAD supplementation). A variety of animal or human pathogens, such as *Streptococcus mutans*, *Pseudomonas aeruginosa*, *Legionella pneumophila*, *Stenotrophomonas maltophilia*, and *Escherichia coli*, have been reported that to form mixed biofilms, which help to improve their resistance, persistence, and pathogenicity (Yang et al., 2011). Recent studies on multi-species community behavior have shown that the presence of other bacterial species can modulate the virulence and the gene expression of pathogens. In the respiratory tract, the NAD supply, which is essential for *A. pleuropneumoniae* growth, is rather limited. However, *A. pleuropneumoniae* has overcome this deficiency in various ways, such as cell lysis, which releases nutrients into the surrounding environment (Chiers et al., 2010). In addition, polymicrobial biofilm formation with *S. suis* enables *A. pleuropneumoniae* to acquire this compound by cross-feeding.

We showed that a mixed biofilm of *S. suis* and *A. pleuropneumoniae* has an increased resistance to several antibiotics. In fact, it is well known that bacteria residing in biofilms have an increased resistance to antibiotics (Assavacheep and Rycroft, 2013), and multi-species biofilm is believed to provide an enhanced protection against antibiotic and host immune system (Armbruster et al., 2010). Some well-known mechanisms for enhancing multi-microbial biofilm resistance include upregulation and transfer of drug resistance genes, increased responsiveness through quorum sensing, and increased mutation level in antibiotic target molecules (Hoiby et al., 2010). Recent research of multi-species community behavior have reported that the virulence, biofilm formation, and the gene expression level of pathogens can be regulated by the presence of other species (Duan et al., 2003). To investigate this, quantitative PCR was performed to analyze differential gene expression in mixed biofilms. The RT-PCR results showed that genes of *A. pleuropneumoniae*, coding for exotoxin, biofilm formation, or iron uptake, respectively, were highly induced. Further, genes of *cps2*, *gdh*, *mrp*, and *sly* associated with virulence factors of *S. suis* were significantly upregulated. The result suggests that mixed biofilms may reinforce bacteria pathogenicity.

In conclusion, we found that co-culture may result in increased antibiotic resistance and upregulated virulence gene expression for *S. suis* and *A. pleuropneumoniae* in biofilms. It is likely that the interspecies interactions between *S. suis* and *A. pleuropneumoniae* are synergetic under specific conditions. Therefore, the interactions between the species in the biofilm community potentially influence the clinical course of disease. Our findings provide some relevant information that may affect the choice of antibiotics, and shed light on a new perspective for the treatment of mixed infection.

## Data Availability Statement

All datasets generated for this study are included in the article/supplementary material.

## Author Contributions

YW and LY conceived and designed the experiments. SG, XD, and JL performed the experiments. YW, XD, and DG analyzed the data. LY and JL contributed reagents, materials, and analysis tools. YW and SG wrote the manuscript.

## Conflict of Interest

The authors declare that the research was conducted in the absence of any commercial or financial relationships that could be construed as a potential conflict of interest.
